# Phase I Study of Simlukafusp Alfa (FAP-IL2v) with or without Atezolizumab in Japanese Patients with Advanced Solid Tumors

**DOI:** 10.1158/2767-9764.CRC-24-0185

**Published:** 2024-09-06

**Authors:** Takafumi Koyama, Kan Yonemori, Toshio Shimizu, Jun Sato, Shunsuke Kondo, Kazuki Sudo, Tatsuya Yoshida, Yuki Katsuya, Tatsuki Imaizumi, Masashi Enomoto, Ryoko Seki, Noboru Yamamoto

**Affiliations:** 1 Department of Experimental Therapeutics, National Cancer Center Hospital, Tokyo, Japan.; 2 Department of Medical Oncology, National Cancer Center Hospital, Tokyo, Japan.; 3 Department of Pulmonary Medicine and Medical Oncology, Graduate School of Medicine, Wakayama Medical University, Wakayama, Japan.; 4 Department of Hepatobiliary and Pancreatic Oncology, National Cancer Center Hospital, Tokyo, Japan.; 5 Department of Thoracic Oncology, National Cancer Center Hospital, Tokyo, Japan.; 6 Chugai Pharmaceutical Co., Ltd, Tokyo, Japan.

## Abstract

**Purpose:**

The aim of the study was to evaluate the safety/tolerability and pharmacokinetics of simlukafusp alfa (FAP-IL2v), an immunocytokine containing an anti-fibroblast activation protein-α (FAP) antibody and an IL2 variant, administered alone or with the PDL1 inhibitor atezolizumab, in Japanese patients with advanced solid tumors.

**Patients and Methods:**

In this phase 1, open-label, dose-escalation study, patients received i.v. FAP-IL2v at 10 or 15/20 mg alone or 10 mg when combined with i.v. atezolizumab. The primary objectives were identification of dose-limiting toxicities (DLT), recommended dose, and maximum tolerated dose, and evaluation of the safety/tolerability and pharmacokinetics of FAP-IL2v alone and combined with atezolizumab.

**Results:**

All 11 patients experienced adverse events (AE) during FAP-IL2v treatment. Although most AEs were of mild severity, four treatment-related AEs led to study treatment discontinuation in two patients: one with infusion-related reaction, hypotension, and capillary leak syndrome, and the other with increased aspartate aminotransferase. No AE-related deaths occurred. One DLT (grade 3 hypotension) occurred in a patient receiving FAP-IL2v 15/20 mg alone. The recommended dose and maximum tolerated dose could not be determined. The pharmacokinetics of FAP-IL2v remained similar with or without atezolizumab. The study was terminated early as FAP-IL2v development was discontinued because of portfolio prioritization (not for efficacy/safety reasons).

**Conclusions:**

This study describes the safety/tolerability of FAP-IL2v 10 mg alone and in combination with atezolizumab in Japanese patients with advanced solid tumors; one DLT (hypotension) occurred with FAP-IL2v 15/20 mg. However, dose escalation of FAP-IL2v was not conducted because of early study termination.

**Significance:**

This phase I study assessed the safety/tolerability and PK of simlukafusp alfa alone or combined with atezolizumab in Japanese patients with advanced solid tumors. No notable differences in PK were noted with the combination versus simlukafusp alfa alone; however, high-dose simlukafusp alfa treatment was associated with recombinant IL2-related toxicity, despite the drug's FAP targeting and IL2Rβγ-biased IL2 variant design.

## Introduction

IL2-based immunotherapy for cancer is complex. IL2 can stimulate the immune response by promoting the proliferation, growth, and activation of T, B, and NK cells (1, 2). However, IL2-based immunotherapy presents several challenges. First, IL2 controls excessive immune and autoimmune responses by promoting regulatory T cell (Treg) expansion, including CD4^+^/CD25^+^ Treg expansion (3) and maintenance of these cells at maturity (4), and via Fas-mediated apoptosis of activated T cells (i.e., activation-induced cell death; ref. 5). Therefore, IL2-based immunotherapy may have the paradoxical effect of dampening antitumor immune responses. Second, treatment with recombinant IL2 may be associated with severe adverse effects, including capillary leak syndrome (vascular leak syndrome); severe infection, systemic inflammatory response syndrome, or pyrexia (fever); and cardiovascular, gastrointestinal (GI), hematologic, neurologic, hepatic, or urogenital adverse events (AE). At present, the only wild-type (WT) recombinant IL2 cancer treatment approved for usage is aldesleukin, which is available for metastatic melanoma or renal cell carcinoma (RCC) in several countries worldwide (6).

To overcome the challenges of IL2-based immunotherapy, a recombinant variant IL2 (IL2v) was developed with the aim of abrogating the binding of IL2 to the α-subunit of the IL2 receptor (IL2Rα) while retaining binding to the β- and γ-subunits of IL2R (IL2Rβγ), thereby preventing biased expansion of Tregs over immune effector cells (7, 8). To facilitate the retention of IL2v in the tumor microenvironment, IL2v was fused to an antifibroblast activation protein-α (FAP) antibody (FAP-IL2v), and the resulting immunocytokine was named simlukafusp alfa (formerly RO6874281/RG7461; ref. 7).

FAP is a type II transmembrane glycoprotein, with proteolytic activity rarely expressed in healthy adult tissue (9) but overexpressed on the surface of cancer-associated fibroblasts of several tumor types, including the breast, esophagus, lung, pancreas, colon, and head and neck (10, 11). In a murine model of FAP-expressing B16 melanoma tumors, FAP-IL2v treatment induced strong CD8^+^ T- and NK-cell expansion, consistent with its expected pharmacodynamic effects (12). In such an investigation, two intravenous injections of FAP-IL2v (given 3 days apart) were well tolerated with no apparent severe adverse effects (12). A mild decrease in body weight was observed after drug administration; however, this normalized within 2 days (12). Simlukafusp alfa is intended to be given in combination with cancer immunotherapies that act via immune effector cells because FAP-IL2v increases the population of activated immune effector cells (7).

Cancer immunotherapies designed to target programmed death-1 (PD1)/PDL1 signaling showed antitumor responses in many cancers (including melanoma, non–small cell lung cancer, and various carcinomas); however, successful, durable, and long-lasting therapeutic responses are not experienced by all patients (13). In an attempt to improve response to immunotherapy, researchers evaluated the synergistic antitumor activity of combining a PDL1-blocking agent with a therapy that exhibits an alternative mechanism of action (14). In this regard, results from murine tumor models showed that combining simlukafusp alfa with atezolizumab (a humanized IgG1) mAb against PDL1 (14)] improves the therapeutic effect of atezolizumab, which has been attributed to the enhanced activation of antigen-specific tumor T cells (7). In phase I clinical trials in patients with advanced solid tumors, simlukafusp alfa resulted in expansion and activation of peripheral NK cells and CD8^+^ T cells (not Tregs) in peripheral blood (15, 16), which is consistent with the mechanism of action of simlukafusp alfa. The recommended dosage for intravenous simlukafusp alfa in these phase I trials was identified as 15 mg, followed by 20 mg once weekly using one-step intrapatient escalation, when administered as monotherapy (15), and 10 mg once every 3 weeks as extension therapy following 4 weeks of dose escalation (5–25 mg/week) when administered in combination with atezolizumab plus or minus bevacizumab (16).

The aim of the present study was to investigate the safety, tolerability, and pharmacokinetics (PK) of simlukafusp alfa alone and in combination with atezolizumab in Japanese patients with advanced solid tumors.

## Materials and Methods

### Study design

This phase I, open-label, dose-escalation study was conducted among Japanese patients with advanced solid tumors at National Cancer Center Hospital in Tokyo, Japan. The study was conducted in two stages, during which patients received simlukafusp alfa alone (stage 1) or in combination with atezolizumab (stage 2; Supplementary Fig. S1).

The study protocol was reviewed and approved by the Institutional Review Board of the participating institute (National Cancer Center Hospital in Tokyo, Japan). The study was conducted in accordance with the principles of the Declaration of Helsinki and Good Clinical Practice; written informed consent was obtained from all study participants. The study was registered prospectively with the Japan Registry of Clinical Trials on April 19, 2019 (jRCT2080224653).

### Patients

Eligible patients were males or females ≥20 years of age who presented with histologically or cytologically confirmed advanced or recurrent solid tumors, an Eastern Cooperative Oncology Group performance status of 0 or 1, adequate organ function, measurable disease according to RECIST criteria version 1.1 (17), and an expected survival of at least 12 weeks.

Key exclusion criteria were prior treatment with immune checkpoint inhibitors, anticytotoxic T lymphocyte–associated protein 4 agents, costimulatory agonists, or other cancer immunotherapies (including IL2 therapy) that had to be discontinued because of immune-related adverse reactions or resulted in grade ≥3 immune-related adverse reactions, concomitant or previous autoimmune disease, and significant intercurrent cardiovascular, GI, or pulmonary disorders.

### Treatment

The starting dose of simlukafusp alfa (RO6874281, Chugai Pharmaceutical Co., Ltd.) for this trial was determined using the safety and tolerability data from two international clinical trials, neither of which enrolled Japanese patients (15, 16). Those trials identified a recommended dosage for simlukafusp alfa of 15 mg for the first dose, followed by 20 mg for subsequent doses once weekly for monotherapy and 10 mg once every 3 weeks, when combined with atezolizumab, based on the incidence of dose-limiting toxicity (DLT).

In the first dose cohort of stage 1, three to six patients received simlukafusp alfa at an initial dose of 10 mg (Supplementary Fig. S2), which was slightly lower than the recommended dose (RD) for extension of simlukafusp alfa in the two international clinical trials (15, 16). Simlukafusp alfa was administered once weekly for the first 28-day cycle (cycle 1) and then once every 2 weeks for cycle 2. In the second dose cohort of stage 1, three to six patients received simlukafusp alfa 15 mg on day 1 of cycle 1, followed by 20 mg on day 8 of cycle 1 and beyond. As per the first dose cohort, simlukafusp alfa was administered once weekly for cycle 1 and then once every 2 weeks for cycle 2.

In this study, the simlukafusp alfa dose was escalated using a standard “3 + 3” design, in which patients were evaluated for DLT for 28 days after the initiation of cycle 1 of treatment (the DLT evaluation period). If no DLTs were observed in the first three DLT-evaluable patients, a new cohort was enrolled at the next dose-level cohort. If a DLT was observed in one of the three DLT-evaluable patients, three additional patients were enrolled in the cohort. If no DLTs occurred in the three additional DLT-evaluable patients (i.e., if a DLT was observed in one of the six DLT-evaluable patients), new patients were enrolled at the next dose-level cohort. If a DLT was observed in two or more DLT-evaluable patients, no new patients were enrolled. In addition, the dose-escalation phase of the study was discontinued. If the number of patients enrolled in the previous low-dose-level cohort was three, the addition of patients to the previous cohort was considered in order to confirm additional tolerability.

After confirming the tolerability of simlukafusp alfa 10 mg once weekly in stage 1, patients in stage 2 received simlukafusp alfa at this dosage (administered in the same treatment schedule as the first dose cohort of stage 1) and intravenous atezolizumab 840 mg once every 2 weeks. At each dose level, the treatment was continued until disease progression, a DLT, another AE that would hinder the patient’s participation in the study, or the patient requested discontinuation from the study.

### Outcomes

The primary objectives of this study were to determine the DLT, RD, and MTD, and investigate the safety, tolerability, and PK profiles of simlukafusp alfa alone and in combination with atezolizumab.

DLTs were defined as any AE fulfilling the criteria listed in Supplementary Table S1, which occurred during the DLT evaluation period and was considered causally related to simlukafusp alfa or atezolizumab. Safety and tolerability were assessed by the frequency of AEs, serious AEs (SAE), and DLTs. AEs were classified using the Japanese Medical Dictionary for Regulatory Activities version 22.0, and the severity of AEs was graded according to NCI Common Terminology Criteria for Adverse Events, version 5.0 (18).

Pharmacokinetic outcomes of simlukafusp alfa consisted of the time to maximum observed serum concentration (T_max_), maximum observed serum concentration (C_max_), area under the serum concentration–time curve from time 0 to time of the last measurable serum concentration (AUC_last_), terminal elimination half-life (t_1/2_), and accumulation ratio (calculated using elimination rate constant after a single dose). Next, the T_max_, C_max_, and AUC_last_ of atezolizumab, when administered in combination with simlukafusp alfa, were also determined.

A secondary objective of this study was to determine the antitumor effects in solid tumors as efficacy outcomes of simlukafusp alfa. Efficacy outcomes included the best overall response, progression-free survival (PFS), and duration of response. Patients were considered responders if they had best overall complete response (CR) or partial response (PR). PFS was defined as the period from the start of study drug administration to the date of confirmed disease progression or death, whichever comes first. Among responders, the duration of response was defined as the period from the first CR or PR to the date of confirmed disease progression or death, whichever comes first. Efficacy outcomes were assessed using RECIST version 1.1.

An exploratory analysis of baseline biomarker expression (i.e., FAP and PDL1) was also conducted, whereby expression of FAP and PDL1 in archival tumor samples was assessed by IHC analysis at a central laboratory. FAP staining was conducted using Ventana FAP (SP325) Robust Prototype Assay (Ventana Medical Systems Inc.), in which the FAP positivity was defined as an FAP intensity score of >25 (19). PDL1 staining was conducting using clone 22C3, and the tumor proportional score was calculated.

### Statistical analysis

The planned sample size for the study was 9 to 18 patients in total: 6 to 12 patients in stage 1 (three to six per cohort) and 3 to 6 patients in stage 2 (three to six per cohort). The DLT analysis set included all patients from the safety analysis set who were evaluable for DLTs, whereas safety and efficacy analysis sets included all patients who received ≥1 dose of simlukafusp alfa or atezolizumab. The PK analysis set included all patients who received ≥1 dose of simlukafusp alfa or atezolizumab and whose serum concentration of simlukafusp alfa or atezolizumab had been measured at least once.

Descriptive statistics were calculated and included means, SDs, medians and ranges for continuous variables, and frequencies and proportions for categorical variables. PK parameters were calculated using WinNonlin, version 8.2 (RRID: SCR_024504), and all other statistical analyses were performed using SAS version 9.4 (RRID: SCR_008567).

### Data availability

Source data generated in this study are not publicly available because of the potential compromise to patient privacy. However, derived data supporting the findings of this study may be available from the corresponding author upon request.

## Results

### Patients

**Figure 1:**
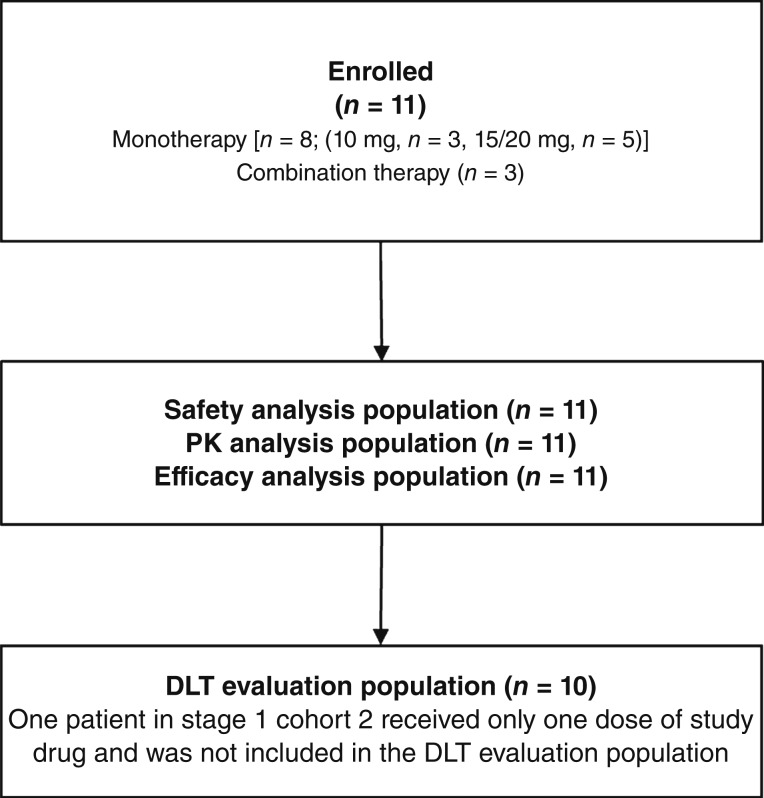
Patient flow.

Between October 15, 2019 and February 17, 2023, 11 patients were enrolled and received simlukafusp alfa [eight patients receiving simlukafusp alfa alone (stage 1) and three receiving simlukafusp alfa plus atezolizumab (stage 2)]. One patient receiving simlukafusp alfa 15 mg and then 20 mg as monotherapy and one patient receiving simlukafusp alfa 10 mg plus atezolizumab discontinued treatment, both because of progressive disease; however, these patients were included in the PK, safety, and efficacy analyses because they met the inclusion criteria for these populations (Fig. 1). Next, 10 patients were included in the DLT population; one in stage 1 cohort 2 received only one dose of simlukafusp alfa was excluded from DLT evaluation.

The histologic diagnoses of the patients were pancreatic, gastric, small intestinal (SI), urachal, thymic, prostate, renal pelvic, and small cell lung cancers (SCLC) and sarcoma. All 11 patients received prior chemotherapy for solid tumors, eight received surgery, and five received radiotherapy (Supplementary Table S2). The representativeness of the study population is shown in Supplementary Table S3.

The median (range) duration of follow-up was 248.0 (30.0–257.0) days for patients who received simlukafusp alfa 10 mg alone (*n* = 3), 94.0 (44.0–828.0) days for those who received simlukafusp alfa 15/20 mg alone (*n* = 5), and 103.00 (66.0–355.0) days for those who received simlukafusp alfa 10 mg plus atezolizumab (*n* = 3).

Patients had a median (range) age of 56.0 (29–71) years, and all were men; the majority of patients (90.9%) had an Eastern Cooperative Oncology Group performance status of 0 (Table 1).

**Table 1:** Baseline characteristics

Characteristic	Simlukafusp alfa 10 mg (*n* = 3)	Simlukafusp alfa 15/20 mg (*n* = 5)	Simlukafusp alfa 10 mg + atezolizumab (*n* = 3)	All (*N =* 11)
Age, years				
Mean ± SD	57.0 ± 11.8	52.8 ± 11.1	52.7 ± 20.8	53.9 ± 12.9
Median (range)	60.0 (44–67)	46.0 (45–71)	61.0 (29–68)	56.0 (29–71)
Age group, years, *n* (%)				
<65	2 (66.7)	4 (80.0)	2 (66.7)	8 (72.7)
≥65	1 (33.3)	1 (20.0)	1 (33.3)	3 (27.3)
Men, *n* (%)	3 (100.0)	5 (100.0)	3 (100.0)	11 (100.0)
Weight, kg				
Mean ± SD	60.27 ± 2.41	78.25 ± 12.57	67.27 ± 6.92	70.35 ± 11.77
Median (range)	60.9 (57.6–62.3)	76.9 (64.7–96.1)	64.1 (62.5–75.2)	64.7 (57.6–96.1)
Height, cm				
Mean ± SD	169.33 ± 4.64	171.82 ± 2.99	175.47 ± 3.97	172.14 ± 4.09
Median (range)	171.60 (164.0–172.4)	171.60 (168.6–176.0)	173.80 (172.6–180.0)	172.40 (164.0–180.0)
ECOG performance status, *n* (%)				
0	2 (66.7)	5 (100)	3 (100)	10 (90.9)
1	1 (33.3)	0	0	1 (9.1)
Time since diagnosis, months	*n* = 2	*n* = 4	*n* = 3	*n* = 9
Mean ± SD	29.7 ± 14.1	97.3 ± 93.3	85.3 ± 89.2	78.3 ± 77.9
Median (range)	29.7 (19.8–39.7)	79.9 (9.9–219.4)	47.8 (20.9–187.1)	41.4 (9.9–219.4)

Abbreviation: ECOG, Eastern Cooperative Oncology Group.

### Safety

The duration of treatment, total dose, and number of treatment cycles for simlukafusp alfa and atezolizumab during this study are presented in Supplementary Table S4.

Simlukafusp alfa 10 mg alone and simlukafusp alfa 10 mg plus atezolizumab were well tolerated; however, the tolerability of simlukafusp alfa 15/20 mg alone could not be confirmed because the study was terminated during enrollment into the simlukafusp alfa 15/20 mg treatment arm because the sponsor discontinued simlukafusp alfa development in 2021 owing to portfolio prioritization (not due to any safety, efficacy, or quality issues). Therefore, the RD and MTD for simlukafusp alfa could not be determined.

During the study period, no AE-related deaths were reported. In total, one DLT (grade 3 hypotension) was reported in a patient receiving simlukafusp alfa 15/20 mg monotherapy; this DLT was considered related to treatment.

Overall, all patients experienced treatment-emergent and -related AEs during the study (Table 2), with 133, 152, and 118 treatment-emergent AEs reported in patients receiving simlukafusp alfa 10 mg monotherapy, simlukafusp alfa 15/20 mg monotherapy, and simlukafusp alfa 10 mg plus atezolizumab 840 mg.

**Table 2:** Summary of AEs

AE,^a^*n* (%)	Simlukafusp alfa 10 mg (*n* = 3)	Simlukafusp alfa 15/20 mg (*n* = 5)	Simlukafusp alfa 10 mg + atezolizumab (*n* = 3)	All (*N =* 11)
Any AE	3 (100.0)	5 (100.0)	3 (100.0)	11 (100.0)
AEs leading to treatment discontinuation	0	2 (40.0)	0	2 (18.2)
AEs leading to dose modification or interruption	0	1 (20.0)	2 (66.7)	3 (27.3)
Treatment-related AEs	3 (100.0)	5 (100.0)	3 (100.0)	11 (100.0)
Treatment-related AEs leading to treatment discontinuation	0	2 (40.0)	0	2 (18.2)
Treatment-related AEs leading to dose modification or interruption	0	1 (20.0)	2 (66.7)	3 (27.3)
SAEs	0	2 (40.0)	1 (33.3)	3 (27.3)
Ileus	0	1 (20.0)	0	1 (9.1)
GI bleeding	0	0	1 (33.3)	1 (9.1)
Diarrhea	0	1 (20.0)	0	1 (9.1)
Capillary leak syndrome	0	1 (20.0)	0	1 (9.1)
SAEs leading to treatment discontinuation	0	1 (20.0)	0	1 (9.1)
SAEs leading to dose modification or interruption	0	0	0	0
Treatment-related SAEs	0	1 (20.0)	0	1 (9.1)
Capillary leak syndrome	0	1 (20.0)	0	1 (9.1)
Deaths	0	0	0	0

a AEs were classified using MedDRA-J version 22.0.

Abbreviation: MedDRA-J, Japanese Medical Dictionary for Regulatory Activities.

Overall, the most common any-grade AEs reported were infusion-related reaction (IRR) and lymphocyte count decreased (11 patients each; 100.0%), aspartate aminotransferase increased and alanine aminotransferase increased (*n* = 9 each; 81.8%), lymphocyte count increased, ƴ-glutamyltransferase increased, platelet count decreased, blood alkaline phosphatase increased and nausea (*n* = 8 each; 72.7%), blood bilirubin increased and rash (*n* = 7 each; 63.6%), decreased appetite (*n* = 5; 45.5%), and hypoalbuminemia, hypophosphatemia, malaise, and anemia (*n* = 3 each; 27.3%). Most AEs were of grade 1 or 2 in severity; however, all 11 patients experienced grade 4 lymphocyte count decreased (Supplementary Table S5).

Two patients experienced an AE leading to study treatment discontinuation; both were receiving simlukafusp alfa 15/20 mg. One experienced a treatment-related grade 3 infusion-related reaction, grade 3 hypotension, and grade 2 capillary leak syndrome after the third dose, and the other experienced treatment-related grade 2 aspartate aminotransferase increased after the first dose. The former patient also developed treatment-related grade 2 IRRs after the first and second doses of simlukafusp alfa; however, neither of these events led to study treatment discontinuation. Three patients (27.3%) experienced five AEs, leading to dose modification or interruption of study medication: one in the simlukafusp alfa 15/20 mg (grade 2 embolism) and two in the simlukafusp alfa 10 mg plus atezolizumab (one had grade 3 aspartate aminotransferase increased, grade 3 alanine aminotransferase increased, and grade 1 bronchitis, and one had a grade 2 IRR) cohorts.

Four SAEs occurred in three patients (27.3%): diarrhea and ileus in one patient receiving simlukafusp alfa 15/20 mg; GI bleeding in one receiving simlukafusp alfa plus atezolizumab; and capillary leak syndrome in one receiving simlukafusp alfa 15/20 mg. Of these, only capillary leak syndrome was considered to be related to treatment (as described above). No significant changes were found in vital signs, physical examination findings, or other test parameters.

### PK

**Figure 2:**
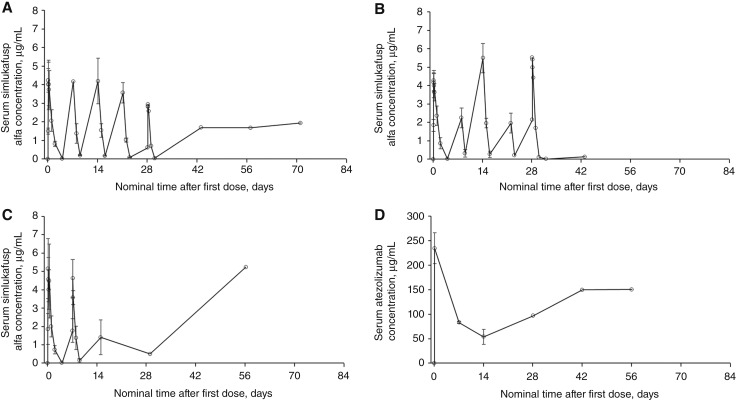
Mean changes in serum simlukafusp alfa concentrations after administration of (**A**) simlukafusp alfa 10 mg (*n =* 3), (**B**) simlukafusp alfa 15/20 mg (*n =* 5), or (**C**) simlukafusp alfa 10 mg + atezolizumab (*n =* 3) and (**D**) mean changes in serum atezolizumab concentrations after administration of simlukafusp alfa 10 mg + atezolizumab (*n =* 3). Error bars represent SD.

After a single-dose infusion of simlukafusp alfa 10 or 15/20 mg, the serum concentration rapidly increased, with a T_max_ of 3.67 and 3.05 hours (time zero was defined as the time of simlukafusp alfa infusion initiation; Table 3). The serum simlukafusp alfa concentration gradually declined, with a t_1/2_ of 9.63 to 12.6 hours. The change in serum simlukafusp alfa concentration over time in patients receiving simlukafusp alfa 10 or 15/20 mg alone is presented in Fig. 2A and B.

**Table 3:** Summary of PK parameters

Parameter	T_max_, hour	C_max_, ng/mL	AUC_last_, μg·hour/mL	t_1/2_, hour
**Simlukafusp alfa**				
Single dose (cycle 1, day 1)				
Simlukafusp alfa 10 mg (*n* = 3)	3.67 (2.17–4.20)	4.41 ± 0.905	115 ± 26.3	12.6 ± 5.01
Simlukafusp alfa 15/20 mg (*n* = 5)	3.05 (2.20–4.00)	4.36 ± 0.508	119 ± 22.5	9.63 ± 0.843
Simlukafusp alfa 10 mg + atezolizumab (*n* = 3)	2.12 (2.12–2.20)	5.15 ± 1.64	116 ± 29.0	10.0 ± 2.18
Multiple doses (cycle 2, day 1)				
Simlukafusp alfa 10 mg (*n* = 2)	NC (2.18–4.03)	3.14 ± NC	46.5 ± NC	6.43 ± NC
Simlukafusp alfa 15/20 mg (*n* = 2)	NC (2.15–3.28)	5.54 ± NC	89.4 ± NC	9.05 ± NC
Multiple doses (cycle 1, day 8)				
Simlukafusp alfa 10 mg + atezolizumab (*n* = 3)	2.10 (2.07–2.18)	4.62 ± 1.03	69.1 ± 17.7	8.87 ± 2.97
**Atezolizumab**				
Single dose (cycle 1, day 1)				
Simlukafusp alfa 10 mg + atezolizumab (*n* = 3)	1.48 (1.43–3.42)	234 ± 31.6	41,700 ± 11,000	NC

Values are presented as mean ± SD or median (range).

Abbreviation: NC, not calculated.

After single and multiple doses of simlukafusp alfa 10 mg plus atezolizumab, the serum simlukafusp alfa concentration increased rapidly, with a T_max_ of 2.10 to 2.12 hours (time zero was defined as the time of simlukafusp alfa infusion initiation). Similar to the PK observed when simlukafusp alfa was administered alone, the serum simlukafusp alfa concentration then gradually declined, with a t_1/2_ of 8.87 to 10.0 hours (Table 3).

The change in serum simlukafusp alfa concentrations over time in patients receiving simlukafusp alfa plus atezolizumab is presented in Fig. 2C. After single doses of simlukafusp alfa 10 mg plus atezolizumab, atezolizumab was rapidly absorbed, with a T_max_ of 1.48 hours (time zero was defined as the time of atezolizumab infusion initiation). The change in serum atezolizumab concentrations over time in patients receiving simlukafusp alfa 10 mg plus atezolizumab is presented in Fig. 2D. The mean ± SD accumulation ratio of simlukafusp alfa was 1.00 ± 1.02 × 10^−3^, 1.00 ± 8.68 × 10^−6^, and 1.00 ± 5.26 × 10^−5^ for patients receiving simlukafusp alfa 10 mg alone, simlukafusp alfa 15/20 mg alone, and simlukafusp alfa 10 mg plus atezolizumab, respectively.

### Efficacy

**Figure 3:**
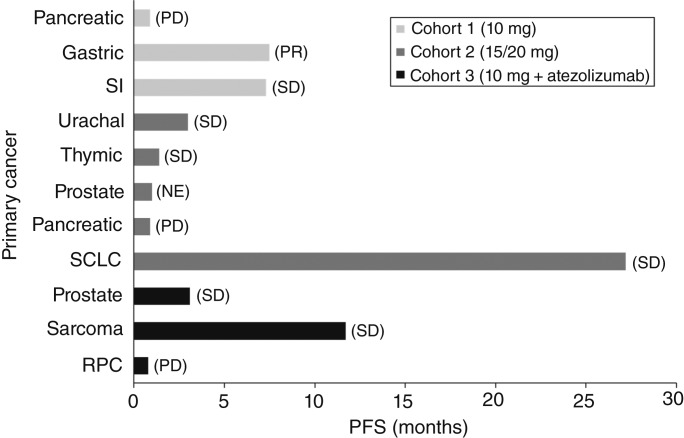
Summary of efficacy with simlukafusp alfa. Best overall response in each patient is presented in parentheses at the end of the bar. NE, not evaluable; PD, progressive disease; RPC, renal pelvis cancer; SD, stable disease.

Of the 11 patients, one patient with gastric cancer receiving simlukafusp alfa 10 mg monotherapy had the best overall response of PR (no confirmation required). This patient previously received immune checkpoint inhibitor (ICI) therapy with single-agent nivolumab as third-line treatment for 95 days. Six patients had stable disease (one receiving simlukafusp alfa 10 mg monotherapy, three receiving simlukafusp alfa 15/20 mg monotherapy, and two receiving simlukafusp alfa 10 mg in combination with atezolizumab), three had progressive disease (one each receiving simlukafusp alfa 10 mg alone, simlukafusp alfa 15/20 mg alone, and simlukafusp alfa 10 mg + atezolizumab), and one (receiving simlukafusp alfa 15/20 mg monotherapy) was not evaluable (Table 4). PFS ranged from 0.9 to 27.2 months. One patient with SCLC refractory to standard chemotherapy, and ICI treatment had a prolonged and sustained disease stabilization (Fig. 3). This patient previously received nivolumab as fourth-line treatment (in combination with an investigational new drug) and had prolonged stable disease for 548 days, despite being refractory to prior standard chemotherapy.

**Table 4:** Summary of efficacy

Variable	Simlukafusp alfa 10 mg			Simlukafusp alfa 15/20 mg					Simlukafusp alfa 10 mg + atezolizumab		
Patient number	1	2	3	4	5	6	7	8	9	10	11
Age, years	44	60	67	46	56	71	46	45	68	29	61
Primary cancer	Pancreatic	Gastric	SI	Urachal	Thymic	Prostate	Pancreatic	SCLC	Prostate	Sarcoma	RPC
BOR	PD	PR	SD	SD	SD	NE	PD	SD	SD	SD	PD
PFS, months	0.9	7.5	7.3	3.0	1.4^a^	1.0^a^	0.9	27.2^a^	3.1	11.7^a^	0.8
DOR, months	–	6.7	–	–	–	–	–	–	–	–	–

a Censored observation.

Abbreviations: BOR, best overall response; DOR, duration of response; NE, not evaluable; PD, progressive disease; RPC, renal pelvis cancer; SD, stable disease.

### Biomarkers

At baseline, FAP and PDL1 expression status was available for 10 of 11 patients (Supplementary Table S6). Four patients were positive for FAP expression [two receiving simlukafusp alfa 10 mg alone (with gastric or SI cancer) and one each receiving simlukafusp alfa 15/20 mg alone (with thymic cancer) and simlukafusp alfa 10 mg + atezolizumab (with sarcoma)], and one with renal pelvic cancer had high PDL1 expression (receiving simlukafusp alfa 10 mg + atezolizumab).

## Discussion

Simlukafusp alfa, administered at 10 or 15/20 mg once weekly/once every 2 weeks alone or 10 mg once weekly in combination with atezolizumab, was associated with one DLT (grade 3 hypotension with 15/20 mg alone) and treatment-emergent and -related AEs (with alone or in combination with atezolizumab) in all 11 patients. Three patients experienced four SAEs, including one patient with treatment-related capillary leak syndrome. Two different recommended doses of simlukafusp alfa were suggested based on preliminary results from two prior phase I studies: 20 mg for monotherapy use from a study of 61 patients with advanced solid tumors (15) and 10 mg for combination therapy with atezolizumab from a study of 69 patients with advanced/metastatic clear-cell and/or sarcomatoid RCC (results presented as an abstract; ref. 16). However, as the study was terminated early, the recommended phase II dose or MTD of simlukafusp alfa could not be determined.

In our study, the patient population was of an age (median 56.0 years) similar to that of the two previous phase I studies (median 57–60 years; refs. 15, 16); however, the histologic diagnoses differed between the studies. Our study included patients with advanced solid tumor types (i.e., pancreatic, gastric, SI, urachal, thymic, prostate, and renal pelvic cancers, SCLC, and sarcoma), whereas the diagnosis was most commonly melanoma or squamous cell carcinoma (SCC) in one of the previous phase I studies (15) and exclusively clear-cell and/or sarcomatoid RCC in the other (16).

In the previous phase I study in 69 patients with metastatic solid tumors, DLTs with simlukafusp alfa included fatigue, asthenia, drug-induced liver injury, transaminase increased, and pneumonia (15). In contrast, our study identified only one DLT (grade 3 hypotension), most likely because of the smaller population size (*n* = 11). The most common AEs reported in our study were IRR, lymphocyte count increased/decreased, liver enzymes and blood bilirubin increased, nausea, decreased appetite, malaise, and anemia. These AEs were consistent with IL2 class effects (20); no FAP-specific AEs were found. The frequency of AEs was consistent with those reported in the abovementioned phase I studies [patients with metastatic solid tumors (15) or advanced/metastatic RCC (16)] and preliminary findings from a phase II study of simlukafusp alfa 10 mg plus atezolizumab 1,200 mg once every 3 weeks in patients with recurrent or metastatic cervical SCC (presented as an abstract; ref. 21). Similar to our study, the IL2 class-specific AEs observed in these previous studies of simlukafusp alfa included pyrexia, anemia, transaminase increase or abnormal liver function tests, edema, and IRRs (15, 16, 21). The IL2 class effects were expected with simlukafusp alfa as it was administered as an IV infusion, and the IL2v moiety is always active and accessible to IL2R-positive cells, regardless of whether or not it is fused to FAP (7). Although IL2v abrogates IL2Rα/CD25 binding compared with WT IL2, IL2v retains full agonistic binding to IL2Rβγ, causing downstream activation of IL2Rβγ-expressing immune cells (e.g., NK and T cells) and secondary release of inflammatory cytokines, including WT IL2. The fusion of an anti-FAP antibody to IL2v allows for increased localized IL2v exposure in FAP-positive tissues in the tumor microenvironment; however, free or unbound FAP-IL2v can still bind to IL2Rβγ in other tissues, causing IL2-related adverse effects (7). Therefore, simlukafusp alfa is expected to demonstrate systemic cytokine activity that is on-target, but off-tumor. However, the overall safety profile of simlukafusp alfa was improved compared with the historic safety profile of aldesleukin, a recombinant IL2 therapy (22). Furthermore, despite high systemic IL2v exposure, only one patient in our study experienced a treatment-related SAE (capillary leak syndrome) with simlukafusp alfa 15/20 mg alone. This disorder is a known cytokine-related AE with aldesleukin treatment (22).

Across the dose range, serum simlukafusp alfa concentrations showed rapid increases after IV administration. Close to dose-proportional elimination of simlukafusp alfa occurred when administered alone after multiple, but not single, doses, consistent with nonlinear clearance that is typical of the target-mediated drug disposition, as previously described in the phase I study in patients with metastatic solid tumors (15). In the prior phase I study of patients with metastatic solid tumors, these nonlinear PK properties were attributed to saturable target-mediated clearance via binding to IL2R (15). No notable difference was observed in the PK profile of simlukafusp alfa when administered with atezolizumab. With regard to pharmacodynamics, although flow cytometry analyses of immune cell populations in the tumors or in peripheral blood were not conducted in this study, both prior phase I trials confirmed that simlukafusp alfa at doses of 10 or 20 mg induced rapid expansion of CD8^+^ and NK cells, but not Tregs (15, 16).

Given that FAP is overexpressed on the surface of cancer-associated fibroblasts, the FAP-mediated retention of simlukafusp alfa in the tumor microenvironment is expected to provide improved antitumor activity (11). An imaging study in patients with advanced solid carcinoembryonic antigen (CEA)–positive tumors previously confirmed selective and targeted biodistribution of another IL2v fusion molecule (the CEA-IL2v agent cergutuzumab amunaleukin) in CEA-positive tumor cells (23). This indicates that patients with FAP-positive tumors may experience improved treatment response to simlukafusp alfa compared with those with FAP-negative tumors. However, treatment efficacy was not a primary objective of this study, and FAP status was not prospectively assessed. No firm conclusions about potential efficacy of simlukafusp alfa can be drawn, given the small study population, nor whether it enhances the efficacy of atezolizumab, given the lack of an atezolizumab monotherapy arm. Furthermore, all treatment regimens in this study included patients with a best response of stable disease or progressive disease, with only one patient achieving a PR (this patient had advanced gastric cancer and was receiving simlukafusp alfa 10 mg alone) and long-term stable disease observed in another patient (this patient presented with SCLC and was receiving simlukafusp alfa 15/20 mg alone). Both patients previously received the ICI treatment with nivolumab (either as monotherapy or in combination with an investigational new drug, respectively), indicating that gastric cancer and SCLC may respond to immunotherapy. Indeed, in the phase III ATTRACTION-2 study in patients with advanced gastric or gastroesophageal junction cancer, third-line nivolumab therapy was associated with a median OS and PFS of 5.26 and 1.61 months, respectively (24). In the previous phase III study in patients with metastatic solid tumors, 1 of 59 evaluable patients had CR, 2 had PR, and 13 had stable disease {objective response rate 5.1% [90% confidence interval (CI), 2.05%–12.06%]; disease control rate 27.1%; ref. 15}. According to the preliminary results from the aforementioned phase II trial in patients with recurrent/metastatic cervical SCC, simlukafusp alfa plus atezolizumab combination therapy was associated with an objective response rate of 27% (90% CI, 18%–39%) and durable response reported [median (95% CI) duration of response 13.3 (7.6–14.7) months; ref. 21]. However, this phase II trial was terminated early because of a commercial decision by the sponsor (unrelated to safety or efficacy; ref. 25).

The main limitations of the current phase I study were the small, heterogeneous, partly imbalanced study population of 11 patients, and the early termination of the study; however, this study exhibited a primary objective of examining the safety of simlukafusp alfa monotherapy or combination therapy and was not designed as a signal-seeking phase Ib or phase II study. Another limitation was the lack of an atezolizumab single-agent treatment arm; however, the safety profile of atezolizumab monotherapy is well established (26) and could be used to make a historic comparison with simlukafusp alfa plus atezolizumab combination therapy. Finally, the results may not be generalizable to other patient populations, including patients with poorer performance status or a different treatment history, or patients of other ethnic groups.

## Conclusions

This phase I study describes the safety and tolerability of simlukafusp alfa as 10 mg once weekly/once every 2 weeks monotherapy and as 10 mg once weekly in combination with atezolizumab in Japanese patients with advanced solid tumors. One DLT (hypotension) was observed with simlukafusp alfa 15/20 mg once weekly/once every 2 weeks monotherapy; however, dose escalation of simlukafusp alfa 15/20 mg could not be confirmed as study enrollment was prematurely terminated. Low-dose simlukafusp alfa was associated with mild treatment-related AEs that were self-limiting and managed without the need for hospitalization; no related neurologic or skin toxicities were observed. Simlukafusp alfa had potentially encouraging antitumor activity; however, as of December 2023, simlukafusp alfa is no longer in development for the treatment of cancer.

## Supplementary Material

Supplementary Figure 1 Figure S1 shows the study design.

Supplementary Figure 2 Figure S2 shows the study progression and dose steps.

Supplementary Table 1 Table S1 shows the adverse events that could be considered dose-limiting toxicities.

Supplementary Table 2 Table S2 shows the prior treatment regimens in the individual study participants.

Supplementary Table 3 Table S3 shows the representativeness of study participants.

Supplementary Table 4 Table S4 shows the duration of treatment and dose.

Supplementary Table 5 Table S5 shows the adverse events by primary system-organ-class and preferred term, overall and those of Grade ≥3 severity.

Supplementary Table 6 Table S6 shows the biomarker expression status at baseline.
