# Supplementary material for: Phase I Study of Simlukafusp Alfa (FAP-IL2v) with or without Atezolizumab in Japanese Patients with Advanced Solid Tumors
Source: Cancer Res Commun. 2024 Sep 6;4(9):2349–58. doi: 10.1158/2767-9764.CRC-24-0185 (PMC11377867; doi:10.1158/2767-9764.CRC-24-0185)
Supplement: Supplementary Figure 1 — Figure S1 shows the study design. [file crc-24-0185_supplementary_figure_1_suppsf1.pdf]

Supplementary Figure S1 Study design. The study was conducted in two stages, during which patients received simlukafusp alfa alone (Stage 1) or in combination with atezolizumab (Stage 2).

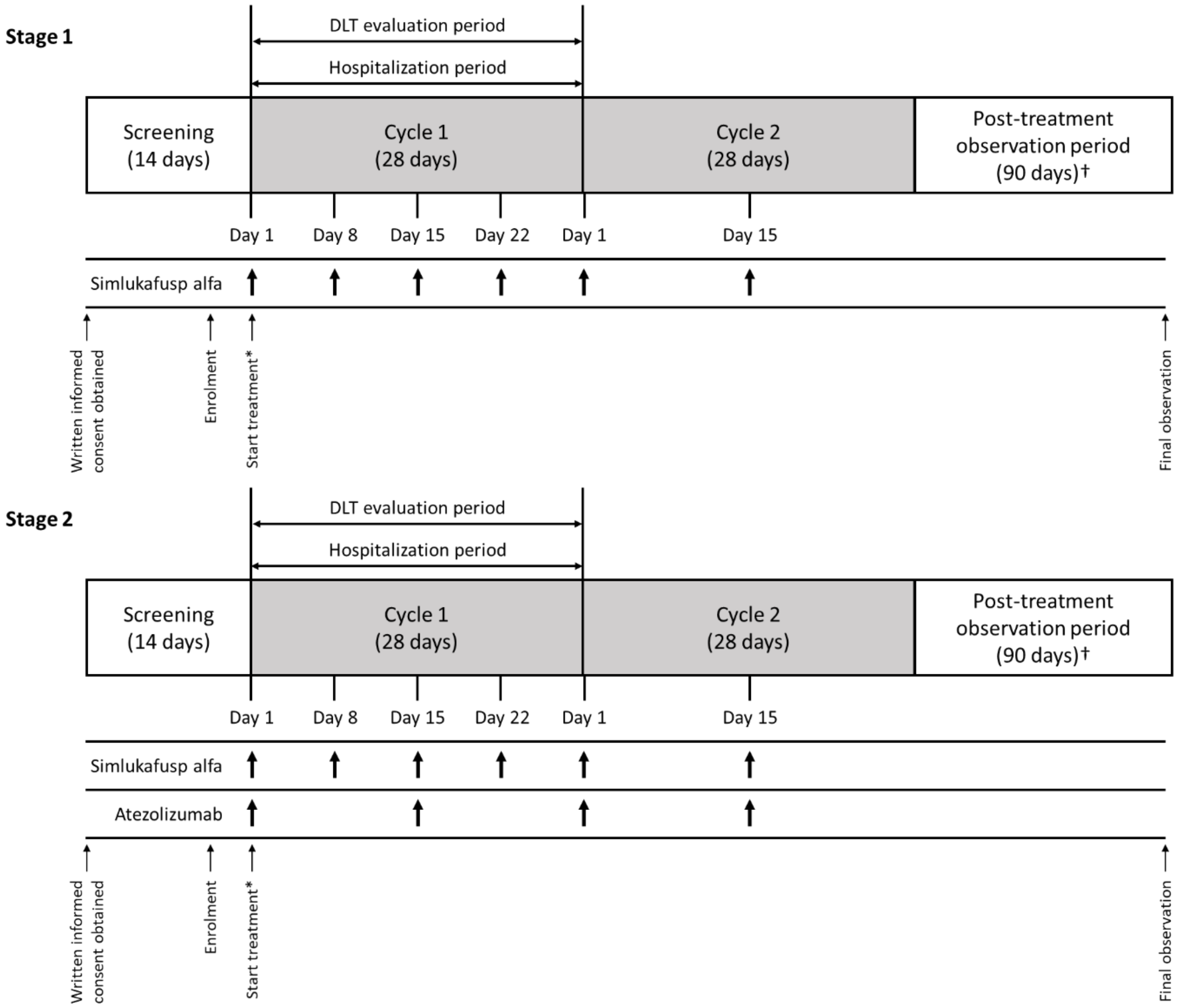

*Treatment was started within 7 days of patient enrollment.
†Treatment was administered until criteria for discontinuation were met (disease progression, DLT or other AE that would hinder the patient's participation in the study) or until the patient requested discontinuation from the study. A final observation was made at 90 ± 7 days after the last dose.
AE, adverse event; DLT, dose-limiting toxicity.
