# Supplementary material for: Phase I Study of Simlukafusp Alfa (FAP-IL2v) with or without Atezolizumab in Japanese Patients with Advanced Solid Tumors
Source: Cancer Res Commun. 2024 Sep 6;4(9):2349–58. doi: 10.1158/2767-9764.CRC-24-0185 (PMC11377867; doi:10.1158/2767-9764.CRC-24-0185)
Supplement: Supplementary Figure 2 — Figure S2 shows the study progression and dose steps. [file crc-24-0185_supplementary_figure_2_suppsf2.pdf]

Supplementary Figure S2 Study progression and dose steps. In the first dose cohort of Stage 1, three to six patients received simlukafusp alfa at an initial dose of 10 mg, which was slightly lower than the recommended dose for extension of simlukafusp alfa in the two international clinical trials.

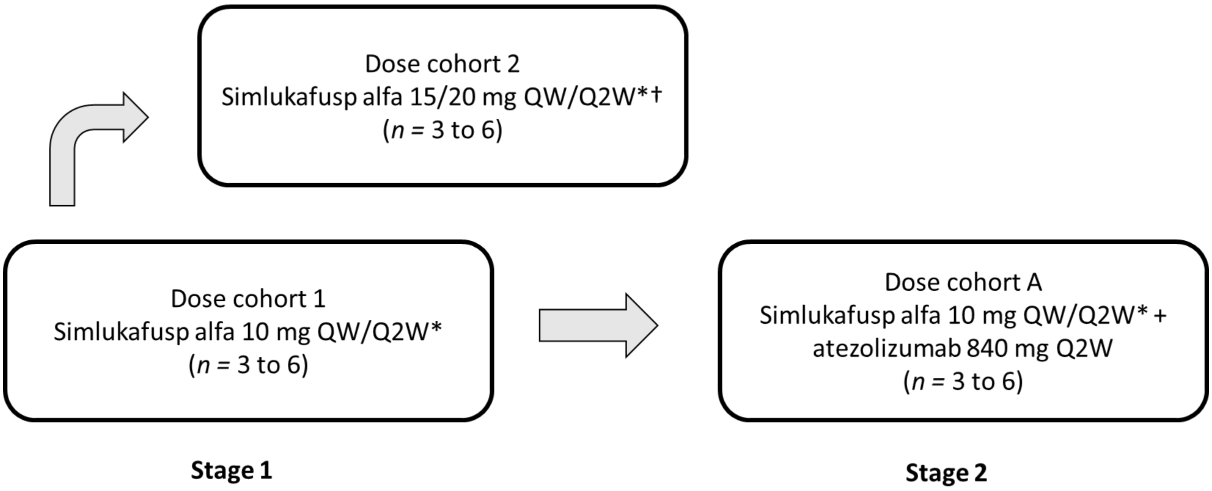

*One cycle was 28 days, with treatment administered QW in Cycle 1 and Q2W in Cycle 2.
†15 mg on Cycle 1 Day 1, then 20 mg on Cycle 1 Day 8 and thereafter.
QW, once weekly; Q2W, once every 2 weeks.
