# Supplementary material for: Phase I Study of Simlukafusp Alfa (FAP-IL2v) with or without Atezolizumab in Japanese Patients with Advanced Solid Tumors
Source: Cancer Res Commun. 2024 Sep 6;4(9):2349–58. doi: 10.1158/2767-9764.CRC-24-0185 (PMC11377867; doi:10.1158/2767-9764.CRC-24-0185)
Supplement: Supplementary Table 1 — Table S1 shows the adverse events that could be considered dose-limiting toxicities. [file crc-24-0185_supplementary_table_1_suppst1.pdf]

SUPPLEMENTARY TABLE S1 Adverse events that could be considered dose-limiting toxicities.

1. Grade ≥3 non-hematologic toxicity and non-hepatic toxicity, excluding:
   a. Grade 3 nausea, vomiting, and diarrhea that resolved to Grade 2 or lower within 3 days (with or without treatment for the event)
   b. Grade 3 fatigue that resolved to Grade 2 or lower within 7 days
   c. Grade 3 pyrexia (>40°C) that resolved within 24 hours
   d. Grade 3 tumor flare (local pain, irritation, or rash in areas where a tumor was present or suspected to be present), which resolved to Grade ≤2 within 7 days
   e. Grade ≥3 laboratory abnormalities that were asymptomatic and judged by the physician to be not clinically significant and resolved to Grade ≤2 within 7 days
   f. Grade 3 skin eruptions that resolved to Grade ≤2 within 7 days with appropriate measures
   g. IRR
2. Grade 4 neutropenia persisting for >7 days or requiring treatment with G-CSF
3. Febrile neutropenia
4. Grade ≥4 anemia requiring transfusion
5. Grade 4 or Grade 3 thrombocytopenia requiring transfusion
6. AST or ALT >3 times the ULN or total bilirubin >2 times the ULN
7. AEs related to simlukafusp alfa or atezolizumab that required deferral of simlukafusp alfa or atezolizumab >14 days in Cycle 1

AEs, adverse events; ALT, alanine aminotransferase; AST, aspartate aminotransferase; G-CSF, granulocyte-colony stimulating factor; IRR, infusion-related reaction; ULN, upper limit of normal.
