# Supplementary material for: Phase I Study of Simlukafusp Alfa (FAP-IL2v) with or without Atezolizumab in Japanese Patients with Advanced Solid Tumors
Source: Cancer Res Commun. 2024 Sep 6;4(9):2349–58. doi: 10.1158/2767-9764.CRC-24-0185 (PMC11377867; doi:10.1158/2767-9764.CRC-24-0185)
Supplement: Supplementary Table 2 — Table S2 shows the prior treatment regimens in the individual study participants. [file crc-24-0185_supplementary_table_2_suppst2.pdf]

SUPPLEMENTARY TABLE S2 Prior treatment regimens in the individual study participants.

Patient number	Age, years	Primary cancer	Prior treatment	Duration, days	Last treatment (study day)
1	44	Pancreatic cancer	Gemcitabine + nab-paclitaxel	346	−236
			Nivolumab	N/A	N/A
			mFOLFIRINOX	114	−115
			Trametinib + hydroxychloroquine	29	−52
2	60	Gastric cancer	S1	196	−890
			XELOX	292	−587
			Ramucirumab + paclitaxel	217	−355
			Nivolumab	95	−239
			Cisplatin + irinotecan	159	−76
3	67	SI cancer	FOLFOX	N/A	N/A
			FOLFIRI	N/A	−313
			Investigational new drug	220	−38
4	46	Urachal cancer	FOLFOX	318	−122
			Investigational new drug	21	−62
5	56	Thymic cancer	Carboplatin + paclitaxel	N/A	N/A
			S1	57	−49
6	71	Prostate cancer	Goserelin	285	−6,351
			Bicalutamide	180	−6,316
			Bicalutamide	1,866	−2,526
			Goserelin	N/A	N/A
			Bicalutamide	175	−2,100
			Flutamide	49	−2,051
			Dexamethasone	818	−1,233
			Docetaxel	581	−1,233
			Enzalutamide	92	−904
			Abiraterone	101	−804
			Cabazitaxel	636	−169
7	46	Pancreatic cancer	mFOLFIRINOX	195	−87
			Gemcitabine + nab-paclitaxel	29	−31
8	45	SCLC	Cisplatin + etoposide	77	−1,165
			Topotecan	81	−962
			Epirubicin	226	−716
			Irinotecan	36	−653
			Investigational new drug + nivolumab	548	−35
9	68	Prostate cancer	Bicalutamide	932	−4,696
			Leuprorelin	3,490	−662
			Bicalutamide	1,141	−2,828
			Flutamide	91	−2,737
			Chlormadinone	91	−2,646
			Ethinylestradiol	742	−1,904
			Enzalutamide	237	−1,667
			Abiraterone	99	−1,568

			Estramustine	224	−1,344
			Abiraterone	41	−1,303
			Ethinylestradiol	36	−1,267
			Docetaxel	171	−1,096
			Prednisolone	171	−1,096
			Investigational new drug	45	−886
			Cabazitaxel	175	−662
			Prednisolone	175	−662
			Investigational new drug	28	−551
			Leuprorelin	422	−124
			Cabazitaxel	414	−124
			Prednisolone	404	−124
			Leuprorelin	22	−103
			Investigational new drug	43	−46
			Leuprorelin	1	−31
10	29	Sarcoma	Nivolumab	N/A	−286
			Investigational new drug	109	−122
11	61	RPC	Gemcitabine + cisplatin	36	−580
			Pembrolizumab	519	−31
			Paclitaxel + carboplatin	1	−40

FOLFIRI, folinic acid + 5-fluorouracil + irinotecan; FOLFOX, oxaliplatin + folinic acid + 5-fluorouracil; mFOLFIRINOX, modified regimen of oxaliplatin + folinic acid + irinotecan + 5-fluorouracil; RPC, renal pelvis cancer; S1, tegafur/gimeracil/oteracil; SCLC, small cell lung cancer; SI, small intestine; XELOX, capecitabine + capecitabine; N/A, not available.
