# Supplementary material for: Phase I Study of Simlukafusp Alfa (FAP-IL2v) with or without Atezolizumab in Japanese Patients with Advanced Solid Tumors
Source: Cancer Res Commun. 2024 Sep 6;4(9):2349–58. doi: 10.1158/2767-9764.CRC-24-0185 (PMC11377867; doi:10.1158/2767-9764.CRC-24-0185)
Supplement: Supplementary Table 3 — Table S3 shows the representativeness of study participants. [file crc-24-0185_supplementary_table_3_suppst3.pdf]

SUPPLEMENTARY TABLE S3 Representativeness of study participants.

Cancer types/subtypes/stages/conditions	Pancreatic, gastric, small intestinal, urachal, thymic, prostate, small cell lung cancer, sarcoma, and renal pelvis
Considerations related to	
Sex	All enrolled patients were male; except for prostate cancer, the other cancer types generally involve both males and females, but only males were enrolled in the study
Age	Median age was 56.0 years; 3 patients were aged ≥65 years
Race/ethnicity	All patients in this study were Japanese
Geography	All patients in this study were from Japan
Other considerations	None
Overall representativeness of this study	This phase 1 study was conducted to investigate the safety and pharmacokinetics of simlukafusp alfa in Japanese patients with advanced solid tumors; efficacy in all tumor types was not verified because the target population had solid tumors and the number of patients with each cancer type was small
