# Supplementary material for: Phase I Study of Simlukafusp Alfa (FAP-IL2v) with or without Atezolizumab in Japanese Patients with Advanced Solid Tumors
Source: Cancer Res Commun. 2024 Sep 6;4(9):2349–58. doi: 10.1158/2767-9764.CRC-24-0185 (PMC11377867; doi:10.1158/2767-9764.CRC-24-0185)
Supplement: Supplementary Table 4 — Table S4 shows the duration of treatment and dose. [file crc-24-0185_supplementary_table_4_suppst4.pdf]

SUPPLEMENTARY TABLE S4 Duration of treatment and dose.

	**Simlukafusp alfa 10 mg (*n* = 3)**	**Simlukafusp alfa 15/20 mg (*n* = 5)**	**Simlukafusp alfa 10 mg + atezolizumab (*n* = 3)**
Duration of treatment, days	152.00 ± 111.85	184.00 ± 345.74	148.67 ± 169.16
Total dose, mg			
Simlukafusp alfa	133.33 ± 80.83	287.00 ± 476.78	130.00 ± 122.88
Atezolizumab	–	–	8,960.00 ± 9,843.90
Number of treatment cycles			
Simlukafusp alfa	13.33 ± 8.08	14.60 ± 23.84	12.67 ± 11.72
Atezolizumab	–	–	10.67 ± 11.72

Data are mean ± standard deviation.
