# Supplementary material for: Phase I Study of Simlukafusp Alfa (FAP-IL2v) with or without Atezolizumab in Japanese Patients with Advanced Solid Tumors
Source: Cancer Res Commun. 2024 Sep 6;4(9):2349–58. doi: 10.1158/2767-9764.CRC-24-0185 (PMC11377867; doi:10.1158/2767-9764.CRC-24-0185)
Supplement: Supplementary Table 5 — Table S5 shows the adverse events by primary system-organ-class and preferred term, overall and those of Grade ≥3 severity. [file crc-24-0185_supplementary_table_5_suppst5.pdf]

SUPPLEMENTARY TABLE S5 Adverse events by primary system-organ-class and preferred term, overall and those of Grade ≥3 severity.

AEs,[a] *n* (%)<br>Primary system-organ-class<br>Preferred term	Simlukafusp alfa 10 mg (*n* = 3)		Simlukafusp alfa 15/20 mg (*n* = 5)		Simlukafusp alfa 10 mg + atezolizumab (*n* = 3)		All (*N* = 11)	
	**Any grade**	**Grade ≥3[b]**	**Any grade**	**Grade ≥3[b]**	**Any grade**	**Grade ≥3[b]**	**Any grade**	**Grade ≥3[b]**
Any class	3 (100.0)	3 (100.0)	5 (100.0)	5 (100.0)	3 (100.0)	3 (100.0)	11 (100.0)	11 (100.0)
Injury, poisoning, and procedural complications	3 (100.0)	0	5 (100.0)	1 (20.0)	3 (100.0)	0	11 (100.0)	1 (9.1)
Infusion-related reaction	3 (100.0)	0	5 (100.0)	1 (20.0)	3 (100.0)	0	11 (100.0)	1 (9.1)
Investigations	3 (100.0)	3 (100.0)	5 (100.0)	5 (100.0)	3 (100.0)	3 (100.0)	11 (100.0)	11 (100.0)
Lymphocyte count decreased	3 (100.0)	3 (100.0)	5 (100.0)	5 (100.0)	3 (100.0)	3 (100.0)	11 (100.0)	11 (100.0)
AST increased	3 (100.0)	0	4 (80.0)	2 (40.0)	2 (66.7)	2 (66.7)	9 (81.8)	4 (36.4)
ALT increased	3 (100.0)	0	4 (80.0)	0	2 (66.7)	1 (33.3)	9 (81.8)	1 (9.1)
Lymphocyte count increased	3 (100.0)	1 (33.3)	3 (60.0)	0	2 (66.7)	0	8 (72.7)	1 (9.1)
GGT increased	3 (100.0)	0	3 (60.0)	0	2 (66.7)	0	8 (72.7)	0
Platelet count decreased	2 (66.7)	0	4 (80.0)	0	2 (66.7)	0	8 (72.7)	0
Blood ALP increased	3 (100.0)	0	2 (40.0)	0	3 (100.0)	0	8 (72.7)	0
Blood bilirubin increased	1 (33.3)	0	3 (60.0)	0	3 (100.0)	0	7 (63.6)	0
Blood creatinine increased	0	0	1 (20.0)	0	1 (33.3)	0	2 (18.2)	0
Neutrophil count decreased	0	0	1 (20.0)	0	1 (33.3)	1 (33.3)	2 (18.2)	1 (9.1)
Weight loss	1 (33.3)	0	1 (20.0)	0	0	0	2 (18.2)	0
WBC count decreased	0	0	1 (20.0)	1 (20.0)	1 (33.3)	1 (33.3)	2 (18.2)	2 (18.2)
Lipase increased	1 (33.3)	0	0	0	0	0	1 (9.1)	0
Blood CPK increased	0	0	1 (20.0)	0	0	0	1 (9.1)	0
Blood TSH decreased	1 (33.3)	0	0	0	0	0	1 (9.1)	0
Blood LDH increased	0	0	0	0	1 (33.3)	0	1 (9.1)	0
ECG QT prolonged	0	0	0	0	1 (33.3)	0	1 (9.1)	0
Metabolism and nutritional disorders	3 (100.0)	0	5 (100.0)	0	2 (66.7)	0	10 (90.9)	0
Decreased appetite	1 (33.3)	0	2 (40.0)	0	2 (66.7)	0	5 (45.5)	0
Hypoalbuminemia	1 (33.3)	0	2 (40.0)	0	0	0	3 (27.3)	0

Hypophosphatemia	2 (66.7)	0	1 (20.0)	0	0	0	3 (27.3)	0
Hyperglycemia	2 (66.7)	0	0	0	0	0	2 (18.2)	0
Dyslipidemia	0	0	1 (20.0)	0	0	0	1 (9.1)	0
Dehydration	0	0	1 (20.0)	0	0	0	1 (9.1)	0
Hypocalcemia	1 (33.3)	0	0	0	0	0	1 (9.1)	0
Hypomagnesemia	0	0	1 (20.0)	0	0	0	1 (9.1)	0
Gastrointestinal disorders	1 (33.3)	0	4 (80.0)	1 (20.0)	3 (100.0)	1 (33.3)	8 (72.7)	2 (18.2)
Nausea	1 (33.3)	0	4 (80.0)	0	3 (100.0)	0	8 (72.7)	0
Diarrhea	0	0	2 (40.0)	1 (20.0)	0	0	2 (18.2)	1 (9.1)
Stomatitis	1 (33.3)	0	1 (20.0)	0	0	0	2 (18.2)	0
Vomiting	0	0	1 (20.0)	0	1 (33.3)	0	2 (18.2)	0
Ileus	0	0	1 (20.0)	1 (20.0)	0	0	1 (9.1)	1 (9.1)
Gastritis	0	0	0	0	1 (33.3)	0	1 (9.1)	0
Gastrointestinal hemorrhage	0	0	0	0	1 (33.3)	1 (33.3)	1 (9.1)	1 (9.1)
Abdominal distension	1 (33.3)	0	0	0	0	0	1 (9.1)	0
Skin and subcutaneous tissue disorders	2 (66.7)	0	4 (80.0)	0	1 (33.3)	0	7 (63.6)	0
Rash	2 (66.7)	0	4 (80.0)	0	1 (33.3)	0	7 (63.6)	0
Pruritus	0	0	1 (20.0)	0	1 (33.3)	0	2 (18.2)	0
Rash maculo-papular	0	0	1 (20.0)	0	0	0	1 (9.1)	0
Dry skin	1 (33.3)	0	0	0	0	0	1 (9.1)	0
General disorders and administration site conditions	2 (66.7)	0	1 (20.0)	0	1 (33.3)	0	4 (36.4)	0
Malaise	2 (66.7)	0	0	0	1 (33.3)	0	3 (27.3)	0
Face edema	0	0	1 (20.0)	0	0	0	1 (9.1)	0
Pyrexia	0	0	0	0	1 (33.3)	0	1 (9.1)	0
Blood and lymphatic system disorders	1 (33.3)	1 (33.3)	1 (20.0)	0	2 (66.7)	2 (66.7)	4 (36.4)	4 (36.4)
Anemia	0	0	1 (20.0)	0	2 (66.7)	2 (66.7)	3 (27.3)	2 (18.2)
Neutropenia	1 (33.3)	1 (33.3)	0	0	0	0	1 (9.1)	1 (9.1)
Infections and infestations	0	0	1 (20.0)	0	1 (33.3)	0	2 (18.2)	0
Enteritis infectious	0	0	1 (20.0)	0	0	0	1 (9.1)	0
Bronchitis	0	0	0	0	1 (33.3)	0	1 (9.1)	0

Rash pustular	0	0	0	0	1 (33.3)	0	1 (9.1)	0
Vascular disorders	0	0	2 (40.0)	1 (20.0)	0	0	2 (18.2)	1 (9.1)
Embolism	0	0	1 (20.0)	0	0	0	1 (9.1)	0
Hypotension	0	0	1 (20.0)	1 (20.0)	0	0	1 (9.1)	1 (9.1)
Capillary leak syndrome	0	0	1 (20.0)	0	0	0	1 (9.1)	0
Renal and urinary disorders	0	0	2 (40.0)	0	0	0	2 (18.2)	0
Hematuria	0	0	2 (40.0)	0	0	0	2 (18.2)	0
Eye disorders	0	0	0	0	1 (33.3)	0	1 (9.1)	0
Conjunctival hemorrhage	0	0	0	0	1 (33.3)	0	1 (9.1)	0
Cardiac disorders	0	0	0	0	1 (33.3)	0	1 (9.1)	0
Atrial fibrillation	0	0	0	0	1 (33.3)	0	1 (9.1)	0
Neoplasms benign, malignant, and unspecified (including cysts and polyps)	0	0	1 (20.0)	0	0	0	1 (9.1)	0
Tumor pain	0	0	1 (20.0)	0	0	0	1 (9.1)	0

[a]AEs were classified using MedDRA-J version 22.0.

[b]Severity of AEs was classified using NCI CTCAE.

AEs, adverse events; ALP, alkaline phosphatase; ALT, alanine aminotransferase; AST, aspartate aminotransferase; CPK, creatine phosphokinase; CTCAE, Common Terminology Criteria for Adverse Events; ECG, electrocardiogram; GGT, γ-glutamyltransferase; LDH, lactate dehydrogenase; MedDRA-J, Japanese Medical Dictionary for Regulatory Activities; NCI, National Cancer Institute; TSH, thyroid-stimulating hormone; WBC, white blood cell.
