# Supplementary material for: Phase I Study of Simlukafusp Alfa (FAP-IL2v) with or without Atezolizumab in Japanese Patients with Advanced Solid Tumors
Source: Cancer Res Commun. 2024 Sep 6;4(9):2349–58. doi: 10.1158/2767-9764.CRC-24-0185 (PMC11377867; doi:10.1158/2767-9764.CRC-24-0185)
Supplement: Supplementary Table 6 — Table S6 shows the biomarker expression status at baseline. [file crc-24-0185_supplementary_table_6_suppst6.pdf]

SUPPLEMENTARY TABLE S6 Biomarker expression status at baseline.

	Simlukafusp alfa 10 mg			Simlukafusp alfa 15/20 mg					Simlukafusp alfa 10 mg + atezolizumab		
Patient number	1	2	3	4	5	6	7	8	9	10	11
Age, years	44	60	67	46	56	71	46	45	68	29	61
Primary cancer	Pancreatic cancer	Gastric cancer	SI cancer	Urachal cancer	Thymic cancer	Prostate cancer	Pancreatic cancer	SCLC	Prostate cancer	Sarcoma	RPC
FAP intensity score	8	65[a]	165[a]	–	70[a]	5	15	NE	0	43[a]	20
PD-L1 TPS, %	<1.0	<1.0	<0.1	–	<1.0	<1.0	<1.0	<1.0	<1.0	<1.0	>95.0

[a]FAP positive, as defined by an FAP intensity score >25.

FAP, fibroblast activation protein-α; NE, not evaluable; PD-L1, programmed death-ligand 1; RPC, renal pelvis cancer; SCLC, small cell lung cancer; SI, small intestine; TPS, tumor proportional score.
